# Nicotinamide Mononucleotide and Nicotinamide Riboside Reverse Ovarian Aging in Rats Via Rebalancing Mitochondrial Fission and Fusion Mechanisms

**DOI:** 10.1007/s11095-024-03704-3

**Published:** 2024-04-29

**Authors:** Nazli Pinar Arslan, Mesut Taskin, Osman Nuri Keles

**Affiliations:** 1 Department of Histology and Embryology, Faculty of Medicine, Ataturk University, Erzurum, Turkey; 2 Vocational School of Health Services, Bingol University, 12000 Bingol, Turkey; 3 Department of Molecular Biology and Genetics, Faculty of Science, Ataturk University, Erzurum, Turkey

**Keywords:** anti-aging, dynamine-related protein 1, mitochondrial dynamics, NAD^+^ precursor, sirtuins

## Abstract

**Purpose:**

This study examined the effects of nicotinamide mononucleotide (NMN) and nicotinamide riboside (NR) on folliculogenesis and mitochondrial dynamics (fission and fusion mechanisms) in ovaries of middle-aged female rats.

**Methods:**

Experimental groups were young, middle-aged (control), middle-aged + NMN and middle-aged + NR. NMN was administered at a concentration of 500 mg/kg intraperitoneally but NR at a concentration of 200 mg/kg by gavage. Follicle stimulating hormone (FSH) and luteinizing hormone (LH) levels were analyzed by ELISA. Hematoxylin-eosin staining sections were used for histopathological examination and follicles-counting. Expression levels of mitochondrial fission (Drp1, Mff and Fis1) and fusion (Mfn1, Mfn2, Opa1, Fam73a and Fam73b) genes as well as Sirt1 gene were analyzed by RT-PCR. Expression levels of fission-related proteins (DRP1, MFF, FIS1 and SIRT1) were analyzed by Western Blot.

**Results:**

Higher ovarian index, more corpus luteum and antral follicles were detected in NMN and NR groups compared to the control. NMN or NR could rebalance LH/FSH ratio. The control group was determined to possess higher expression levels of fission genes and lower expression levels of fusion genes when compared the young group. In comparison with the control group, both NMN and NR group were found to exhibit less mitochondrial fission but more mitochondrial fussion. Higher gene and protein levels for Sirt1 were measured in NMN and NR groups compared to the control group.

**Conclusion:**

This study reveals that NMN alone or NR alone can rebalance mitochondrial dynamics by decreasing excessive fission in middle-aged rat ovaries, thus alleviating mitochondrial stress and correcting aging-induced folliculogenesis abnormalities.

## Introduction

In eukaryotic cells, fission (dividing of mitochondria, fragmentation) and fusion (merging of two mitochondria) mechanisms, which are also referred to as mitochondrial dynamics are responsible for the controlling of the morphology, distribution, movement, number and activity of mitochondria. These mechanisms participitate in mitochondria-dependent biological processes (lipid homeostasis, calcium homeostasis, and ATP production) in the cells by controlling mitochondrial division and morphology [1, 2]. For example, without fusion, function of mitochondria is significantly reduced, oxygen usage and ATP production are compromised, and cells grow much more slowly [3]. Mitochondrial fission mechanism aids in the removal of detrimented mitochondria via mitophagy and promotes apoptosis under conditions of cellular stress. This mechanism is also involved in critical cellular processes such as synapse formation in neurons and embryonic development [4]. In short, the balance in the frequency of fission and fusion events ensures that the functions in mitochondria and cells are performed correctly. However, the deterioration of this balance, especially excessive increase in fission, causes the lost of mitochondrial membrane potential, decrease of oxidative phosphorylation and ATP production, and activation of pro-apoptotic signals [5–7].

The most important proteins related with mitochondrial fusion are mitofusin-1 (MFN1), mitofusin-2 (MFN2) and optic atrophy 1 (OPA1) [8]. In the performed studies, it has been demonstrated that the proteins known as Mitoguardin-1/2 (MIGA1 and MIGA2) found in the outer membrane of mitochondria in *Drosophila* are also involved in mitochondrial fusion. In vertebrates, including humans, the genes responsible for coding for these proteins are Fam73a and Fam73b, respectively. MFN 1, MFN2, FAM73A and FAM 73B mediate the fusion of the outer membrane, while OPA1 plays role in the fusion of the inner membrane [8–12].

The mitochondrial fission proteins in mammals, including humans are MFF, FIS1, MID49, MID51 and DRP1. Of these proteins, DRP1 (Dynamine-related protein 1) is found in the cytoplasm, while other fission proteins localized in the outer membrane of mitochondria and they function as receptors for the recruitment of DRP-1 to the mitochondrial outer membrane. Especially MFF and FIS1 are the most important receptors of DRP1 [12–15].

Sirtuins are proteins that belong to the NAD^+^-dependent protein deacetylase family that affect a wide variety of cellular processes in humans, including aging and obesity, cell cycle, growth and differentiation, inflammation, apoptosis, and stress response. There are seven different sirtuins (SIRT1-7) in mammals. Furthermore, one of these proteins, SIRT1, indirectly affects mitochondrial fission, namely mitochondrial dynamics [16, 17].

Oxidative stress, which occurs due to excessive ROS accumulation, is considered to be the most important factor of ovarian aging and women infertility, since it deteriorates oocyte quality in the ovaries, promotes granulosa cell apoptosis, boosts the degeneration of the corpus luteum, decreases communication between oocytes and granulosa cells, and inhibits folliculogenesis [18–22]. It has been documented in the literature that ROS-induced oxidative stress increases mitochondrial fission and impairs mitochondrial dynamics during aging in various organs, including ovarian [1, 23–28]. Therefore, rebalance of distrupted mitochondrial dynamics via novel drug therapies may be a solution for reversing of ovarian aging.

Nicotinamide adenine dinucleotide (NAD^+^) acts as a coenzyme in the transport of electrons and hydrogen atoms required for energy production from organic molecules [29]. In the literature, it has been shown that increasing NAD + levels improve mitochondrial function, increase ATP production and reduce mitochondrial stress, and ultimately show a protective effect against aging-induced metabolic disorders [29–31]. Furthermore, in a recent study [32], it was shown that increased NAD^+^ level improves mitochondrial functions and reverses ovarian aging. Nicotinamide mononucleotide (NMN) and nicotinamide riboside (NR) are two important molecules used as precursors in the synthesis of NAD^+^ in cells [31, 33]. NMN reduces mitochondrial stress and showing protective property against neurodegenerative diseases, obesity and diabetes. It also ameliorates aging-related mitochondrial stress, thereby showing anti-aging property [33–36]. Furthermore, the effect of NMN on mammalian fertility was tested in a recent study [27], and the research team showed that exogenous NMN increased NAD^+^ level and improved oocyte quality and ovulation efficiency in aged-mice. However, there is no study yet on how NMN affects mitochondrial fission and fusion mechanisms in aged ovaries. On the other hand, it has been reported that NMN balances mitochondrial dynamics by increasing Sirt1 activity, prevents mitochondrial damage and shows anti-aging property [28, 37]. However, there is no study in the literature on how NMN application influences Sirt1 activity in the ovaries. NR is an alternative form of vitamin B3 and is found in milk. As with NMN, NR functions as a NAD + precursor in cells. This molecule induces mitochondrial biogenesis, increases NAD^+^ production, reduces mitochondrial stress and activates sirtuins. It has been stated that NR has protective properties against obesity and aging, as well as many diseases [38, 39]. However, unlike NMN, the possible beneficial effect of NR against ovarian aging and folliculogenesis problem has not yet been tested in a study. Moreover, as in NMN, there is no study in the literature on how NR affects the mitochondrial fission and fusion mechanisms in ovaries.

Therefore, this study focused on examining whether NAD^+^ precursor molecules (NMN and NR) would reverse ovarian aging and alleviate folliculogenesis problems via rebalancing mitochondrial dynamics (mitochondrial fission and fusion mechanisms) in ovaries of middle-aged rats.

## Materials and Methods

### Animals and Treatment Groups

Wistar albino female rats used in the study were housed in standard cages with free access to food and water (tap water) at 20–24°C for a 12 h/12 h light–dark period. The experimental groups were kept in different cages.

Four experimental groups were designed and each group contained 6 rats. The experimental groups were as follow: young (normal), 2) middle-aged (control), 3) middle-aged + nicotinamide mononucleotide (NMN), and 4) middle-aged + nicotinamide riboside (NR). In the experiments, middle-aged groups included 12-month-old rats (300–350 g) and young group (normal) included 5-month-old rats (200–220 g). The estrous cycle in fertile female rats is a four-day cycle composing of four discernable phases: estrus, metestrus, diestrus, and proestrus. In this study, estrous cycle was evaluated using vaginal smear to ensure regular cyclicity of young and middle-aged rats. Then, among rats showing a persistent estrous pattern, ones in proestrus/estrus were chosen for the experiments.

Before NMN or NR administration, the rats were adapted to laboratory conditions for one week. NMN prepared in PBS was administered intraperitoneally at 500 mg/kg concentration and NR was administered orally at 200 mg/kg concentration by gavage [33]. Both substances were applied for 17 days (a total of 17 doses). The inclusion and exclusion criteria determined for the study were summarized in Table I.

**Table I:** The inclusion and exclusion criteria of the study

Inclusion criteria	Exclusion criteria
Wistar albino female rats were included in the experiments	Rats previously used in another study were not included in the experiments
The middle-aged groups included 12-month-old rats with an average weight of 300–350 g	Rats that died before experiment ends were not included in the analyses
The young group included 5-month-old rats with an average weight of 200–220 g	Rats showing signs/symptoms of illness in their skins, such as redness, cyanosis, icterus, wound, and abscess were not included in the experiments
Among rats showing a persistent estrous pattern, ones in proestrus/estrus were chosen	Rats showing signs/symptoms of illness such as nasal discharge, salivation, sneezing, and dyspnoea were not included in the experiments

### Collecting of Blood Samples and Measurement of Serum Hormone Levels

After 17 days of substance administration, rats were anesthetized with isoflurane on day 18 (after 24 h of the last dose). Blood samples (1 mL) taken from the anesthetized rats with syringe were centrifuged (at 13,000 rpm for 15 min) and the obtained serum fractions were stored at -80°C for subsequent analyses. Follicle stimulating hormone (FSH) and luteinizing hormone (LH) levels were determined by using rat-specific ELISA kits (Elabscience, E-EL-R0391 and E-EL-R0026) according to manufacturer's protocol. After the standard graph was prepared according to the kit protocol, the FSH level of each serum sample was calculated as ng/mL and then the values ​​were converted to mLU/mL by considering the factor "uLU/ml FSH = 0.167 ng/mL FSH". According to the kit protocol, LH concentration was calculated as mLU/mL.

### Measurement of Body Weight and Ovarian Weight

Body weight of rats was assayed on the first day (before substance treatment) and on the 18th day of the study. The ovaries of the anesthetized rats were removed and the ovaries were washed with PBS. Ovary weights of rats were measured on the 18th day of the experimental protocol. The ovary weight index was defined by the following formula: ovary weight index = ovarian weight/body weight. Then, one of the two ovaries from each rat was immediately fixed in 10% formaldehyde for histopathology studies, while the remaining ovaries were immediately frozen at -80°C until used for RT-PCR and Western-Blot analyses.

### Follicle Count in Ovaries

After the ovaries were fixed in 10% formaldehyde for 48 h, they were first washed with water for 3 h. Then, they were subjected to dehydration (treatment with 50%, 60%, 70%, 80%, 96% and 100% alcohol, respectively), clearing (with xylene) and infiltration (treating with paraffin) procedures, respectively. Finally, paraffin tissue blocks for the ovaries were prepared. Sections of 4 μm thickness were prepared from the blocks using a microtome (Leica RM2145). After ovarian sections were placed on the slide, they were kept in an oven at 60°C for 20 min to remove the paraffin and fix the tissue on the slide. Tissue sections fixed on the slide were passed through xylene series for complete removal of paraffin and decreasing alcohol series (100%, 96%, 80% and 70%) for hydration. The sections were washed with distilled water and then stained with hematoxylin. After the excess dye was removed by washing, counterstaining was done with eosin. Then, excess eosin was removed with 96% alcohol and the tissue sections were passed through alcohol and xylene series. Finally, entellan was dropped on the sections and the slides were closed with coverslips and examined under light microscope (Nikon Eclipse i50, Tokyo, Japan).

Each section of each ovary was used for follicle counting. An oocyte surrounded by a partial or complete layer of squamous granulosa cells was named as a follicle. Follicles were classified as antral follicle (more than four layers of granulosa cells containing one or more independent antral spaces or one cumulus granulosa cell layer) and atretic follicle (the presence of apoptotic bodies in the granulosa cell layer, a degenerating oocyte, fragmentation of the oocyte nucleus). The structure which developed immediately following ovulation and was made up of lutein cells was termed as Corpora lutea [40–42]. However, when testing the effect of NMN and NR, the numbers of developed antral follicles, atretic follicles and corpora lutea rather than primordial and primary follicles were taken into account more.

### Analysis of Expression Levels of Mitochondrial Fission and Fusion-Related Genes by RT-PCR

To prepare the cDNA library, firstly, mRNA isolation was performed from rat ovarian tissue. In brief, after a sufficient amount of tissue sample was homogenized in the lysis solution, total RNA was isolated from the tissues using RNA isolation kit (Roche, Germany) according to the kit protocol. Isolated RNAs were then stored at -80°C until cDNA synthesis.

The amount and purity of RNA was checked using a NanoDropTM 2000/2000c spectrophotometer (Thermo). Considering the 260/280 ratios (purity) and concentration values ​​given by the device, cDNA synthesis was started. Complementary DNA synthesis was performed according to the cDNA synthesis kit (Applied Biosystems, USA) protocol. In brief, 10 µL of RNA samples were taken into PCR tubes. The reaction was set up for each RNA sample according to the kit protocol and all procedures were done on ice. All cDNAs were stored at − 20°C for the subsequent stages of the.

The changes in expression levels of target genes (Mfn1, Mfn2, Opa1, Fam73a, Fam73b, Mff, Fis1, Drp1 and Sirt 1) were assayed using real-time polymerase chain reaction (PCR). Relative abundance of mRNA was analyzed using the Taqman Probe Kit (Applied Biosystem, USA) protocol. The reaction mixture without complementary DNA was used as negative control. Expression levels of genes were calculated according to the 2 − ΔΔCT method based on β-actin expression as stated in the literature [43]

### Analysis of Expression Levels of Mitochondrial Fission-Related Proteins by Western Blot

To determine the semi-quantitative expression of SIRT1, FIS1, MFF and DRP1 proteins, 50 mg of the ovarian tissue samples were homogenized in RIPA lysis buffer and the homogenates were then subjected to the centrifugation process (at + 4°C and 13,000 g for 15 min). The total protein content of the supernatants was determined according to the method of Bradford [44].

The homogenate was loaded to 10% polyacrylamide gel. The proteins that were separated according to molecular weight on the SDS gel were transferred onto a polyvinylidene fluoride membrane. Then, membrane was blocked with 5% nonfat dry milk powder in 1 × Tris-buffered saline and 0.05% Tween 20 (TBST) for 1 h at room temperature. Afterwards, the membrane was incubated at 4°C overnight with DRP1, SIRT1, FIS1 and MFF antibodies at 1/1000 dilution and washed 3 times with TBST. It was left to the incubation in the presence of horseradish-coupled secondary antibodies at room temperature for one hour and then was washed with TBST (5 min, five times). The detection was done using an enhanced chemiluminescent (ECL) substrate detection system (Thermo Scientific, SuperSignal West Femto/Pico Maximum Sensitivity Substrate). The chemiluminescence imaging was made using the BioRad ChemiDoc™ Touch Imaging System. Signal was quantified using computer-assisted densitometry (ImageJ). The relative expression quantity of proteins was normalized to that of the respective GADPH bands.

### Statistical Analysis

Experiments were conducted in 3 repetitions, data were given as mean ± standard deviation, and statistical significance was ascertained according to Student's t test. P values ​​less than 0.05 were considered significant. RT-PCR results were evaluated according to the 2-(ΔΔCT) method and p values ​​less than 0.0001 were considered significant. In Western blot analysis, Unpaired t test was applied to the groups, p values ​​less than 0.05, 0.01 and 0.001 were considered significant.

## Results

### Analysis of Serum Hormon Levels

**Fig. 1:**
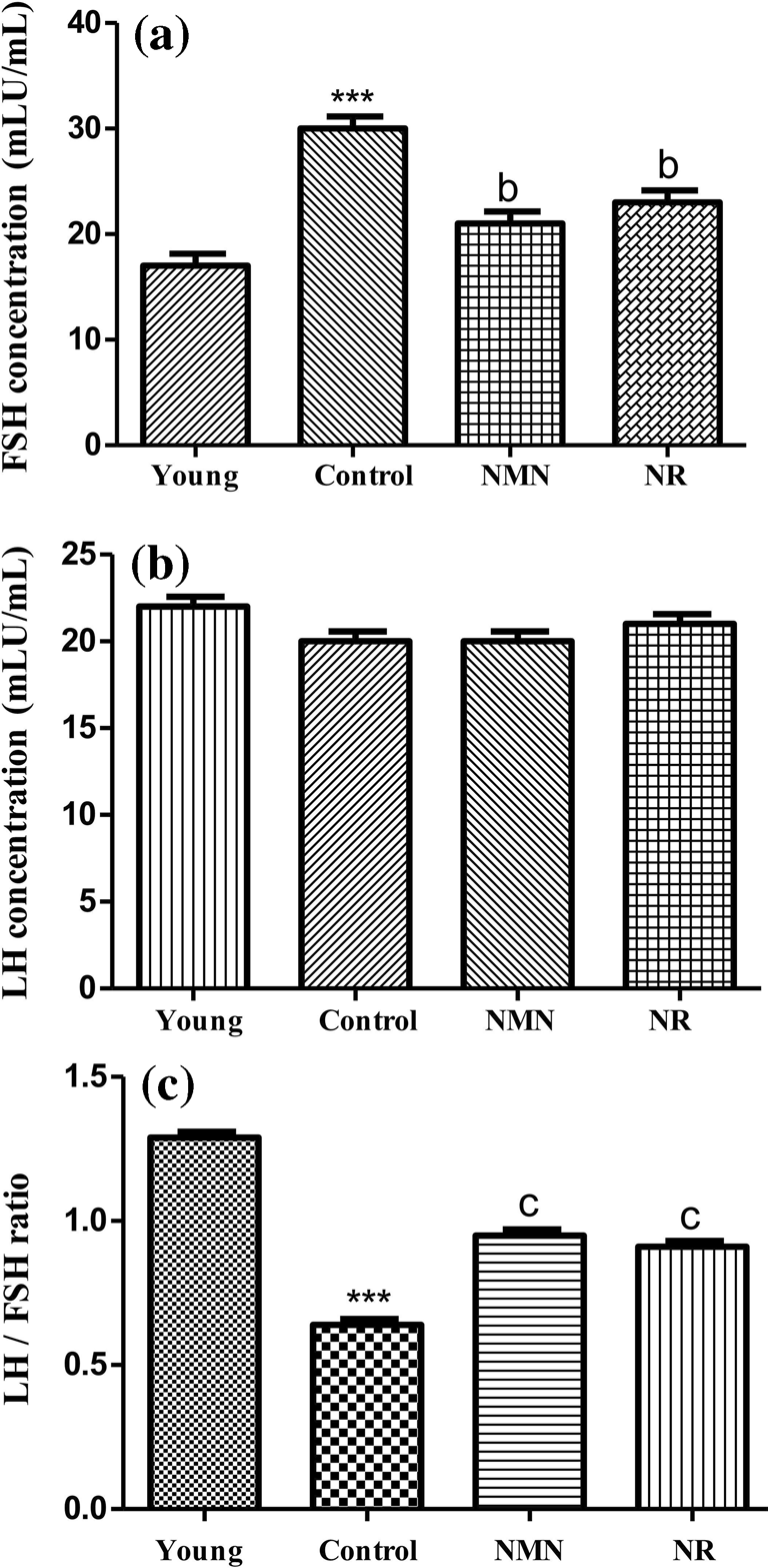
Variation of FSH and LH ratios in experimental groups. Analyses were performed on day 18. Data were calculated as mean ± standard deviation. *** *p* < 0.001 *vs* young, b *p* < 0.01 *vs* control and c *p* < 0.001 *vs* control (a *p* < 0.05, b *p* < 0.01, c *p* < 0.001 and n = 3). Young group included 5 months-rats. Control, NMN and NR groups included middle-aged rats (12 months). Phosphate-buffered saline (PBS) was injected or orally given to the control group. NMN prepared in PBS was administered intraperitoneally at 500 mg/kg concentration (middle-aged NMN group) and NR was administered orally at 200 mg/kg concentration by gavage (middle-aged NMN group). Both NMN and NR was applied for 17 days (total of 17 doses

The experiments revealed that there was a noticable increment in follicle stimulating hormone (FSH) level of the middle-aged control group when compared with the young group (p < 0.05). As compared to the same control group, the significant decreases were detected in the FSH levels of middle-aged NMN and NR groups (p ≥ 0.05). Even, the FSH levels of both two groups decreased approached almost to that of the young group (Fig. 1A). Contrary to FSH levels, luteinizing hormone (LH) values ​​did not show much change in young, control, NMN and NR groups (p ≥ 0.05) (Fig. 1B). LH and FSH values ​​were measured as 22 and 17 mLU/mL in the young group, 20 and 30 mLU/mL in the control group, 20 and 21 mLU/mL in the NMN group and 21 and 23 mLU/mL in the NR group. The LH/FSH ratio was calculated as 1.29, 0.66, 0.95 and 0.91 in the young, control, NMN and NR groups, respectively (Fig. 1C).

### Measurement of Body Weight and Ovarian Weight

**Fig. 2:**
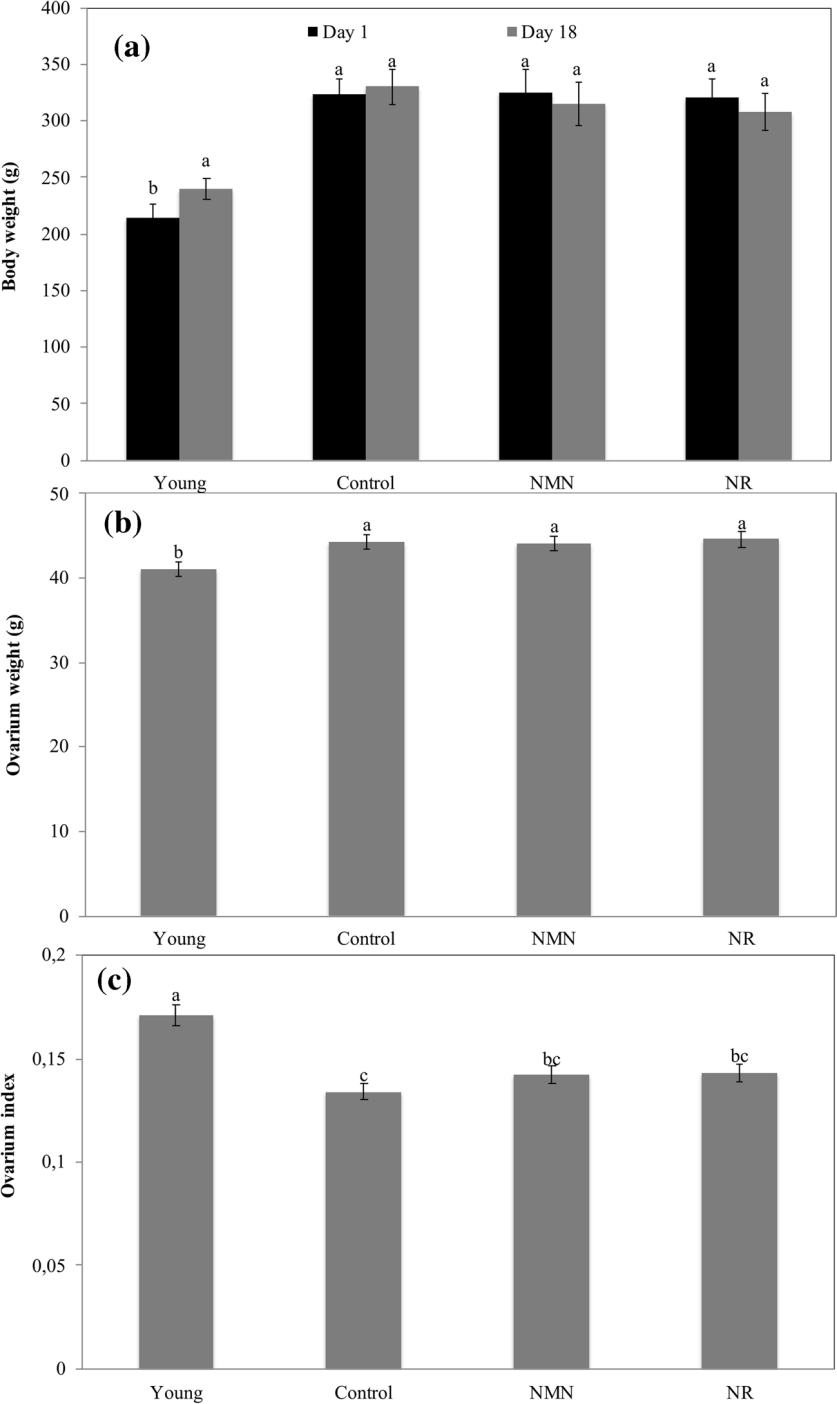
Change of body and ovarian weights in groups. Ovarian weights were measured on day 18. Body weights were measured on days 1 and 18. The values measured for ovarian weight were statistically evaluated among themselves. Similarly, the values measured for body weights were statistically evaluated among themselves. *The difference between the values ​​indicated with the same letters on figure column is not statistically significant (*p* ≤ 0.05).)

At the beginning of the experiments (1st day), average body weight was measured as 215 g for young group, 323 g for middle-aged control group, 320 g for middle-aged NMN group, and 325 g for middle-aged NR group. On the day 18, average body weight was determined as 240 g for young group, 330 g for middle-aged control, 308 g for middle-aged NMN group and 315 g for middle-aged NR group. As seen the the results, the body weight of the control group exhibited a slight increase, while there were slight decreases in the body weights of the NMN and NR groups. However, the differences in the body weights of three groups were not statistically significant (p ≥ 0.05). On the contrary, the results elucidated that the average body weight of the young group increased from 215 to 240 g, and this increase was determined to be statistically significant (p < 0.05) (Fig. 2A).

Avarage ovarian weight at day 18 was measured as 41.1 mg for young group, 44.3 mg for middle-aged control group, 44.1 mg for middle-aged NMN group, and 44.6 mg for middle-aged NR group (Fig. 2B). Ovarian index (ovarian weight/body weight) was calculated as 0.171, 0.134, 0.143 and 0.142 for young, control, NMN and NR groups (Fig. 2C).

### Follicle Count in Ovaries

**Fig. 3:**
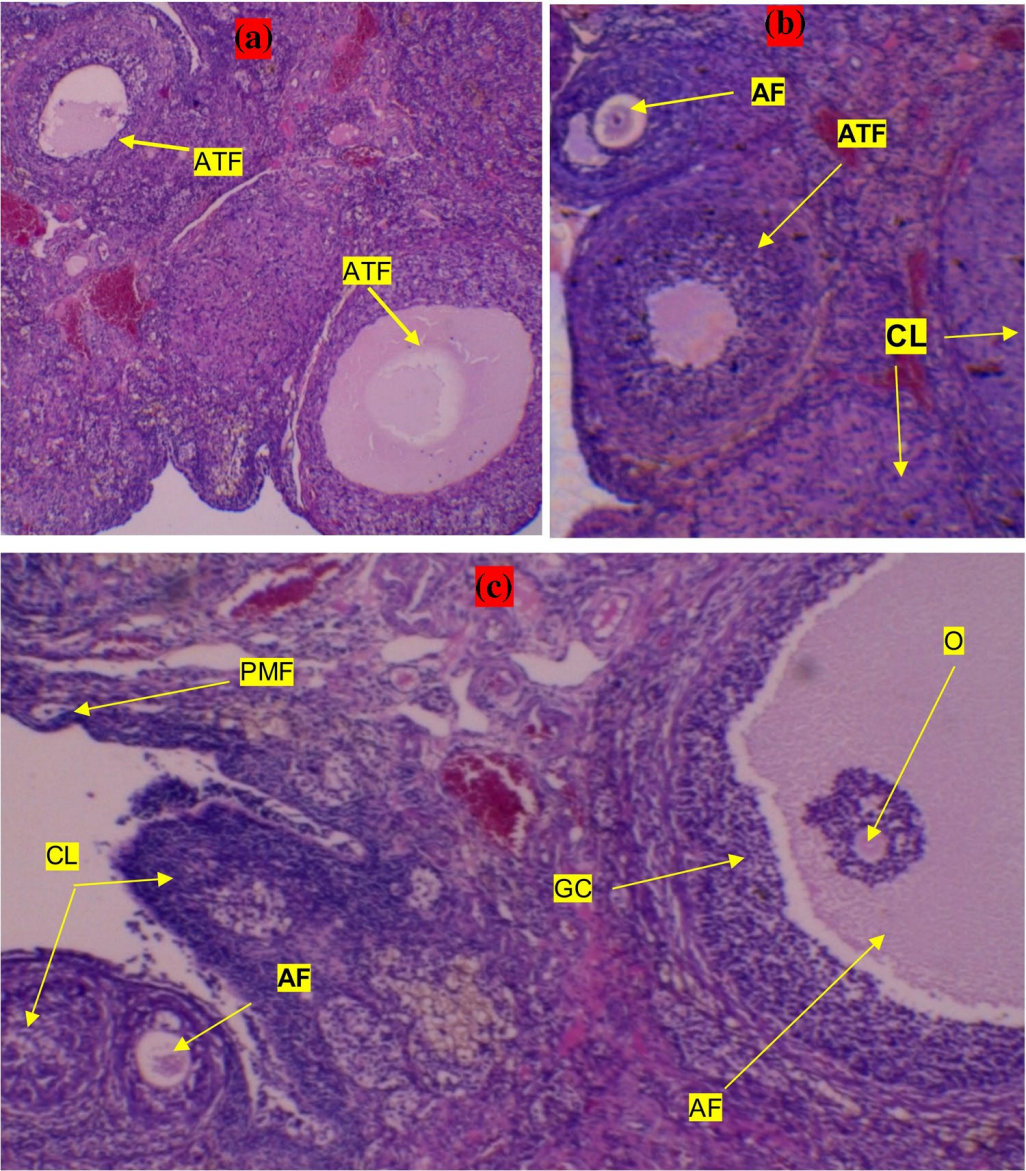
Examination of follicles and corpus luteum in hematoxylin–eosin staining sections. A, B and C indicate control, NR and NMN groups, respectively. PF, primer follicle; PMF, primordial follicle; AF, antral follicle; ATF, atretic follicles; CL, corpus luteum; GC, granulosa cells and O, oocyt. Sections were examined under light microscope (× 10 magnification)

When the hematoxylin–eosin staining sections of the ovaries were examined, it was determined that the ovarian tissues of all groups had a normal appearance. Compared with the middle-aged groups (control, NMN and NR groups), more antral follicles were detected in the ovaries of the young group. As compared with middle-aged control group, both NMN and NR group was found to contain less atretic follicles but more antral follicles and corpus luteum. Atretic follicles were much more prominent especially in the control group (Fig. 3A-C).

**Fig. 4:**
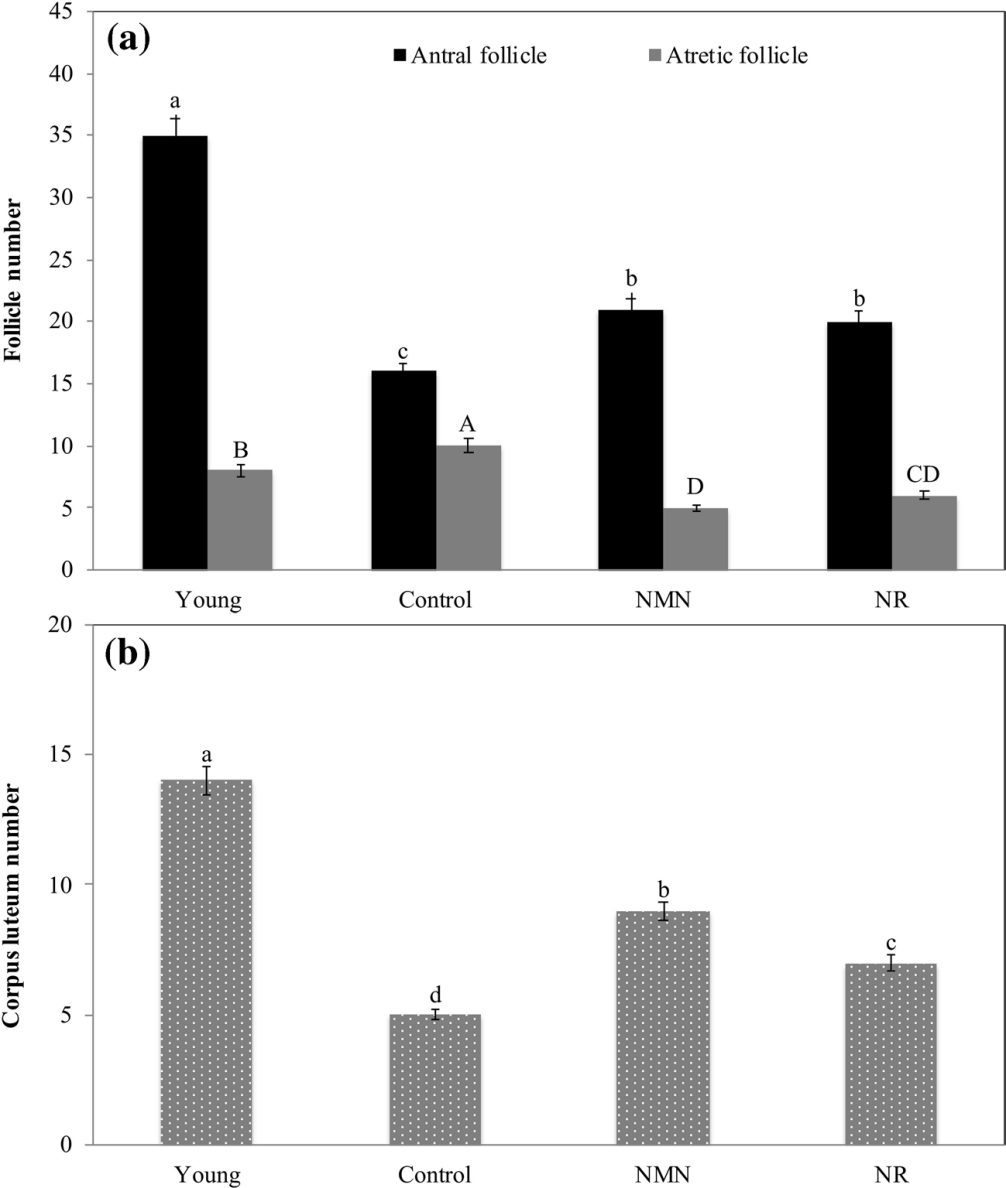
Enumeration of follicles and corpus luteum in treatments groups. Analyses were performed on day 18. Statistical differences in the number of antral follicles are shown in lower letters, whereas statistical differences in the number of atretic follicles are shown in capital letters. The numbers of antral follicles and atretic follicles were not compared statistically with each other. The difference between the values ​​indicated with the same letters on figure column is not statistically significant (*p* ≤ 0.05)

The numbers of antral follicles in the young, control, NMN and NR groups were determined as 35, 16, 21 and 20, respectively. The difference between antral follicle numbers of control group and NMN or NR group was statistically important (p < 0.05) (Fig. 4A). Conversely, the differences between the antral follicle numbers of NMN and NR groups were not statistically important (p ≥ 0.05). As compared with the middle-aged control group, less atretic follicles were detected in the ovaries of NMN and NR groups, and the differences were statistically noticable (p < 0.05). The average numbers of atretic follicles in the ovaries of the young, control, NMN, and NR groups were determined as 8, 10, 5, and 6, respectively (Fig. 4A). Antral follicle/atretic follicle ratio was calculated as 4.3, 1.6, 4.2 and 3.3 for young, control, NMN and NR groups, respectively.

The corpus luteum numbers of the middle-aged NMN and NR groups were higher than that of the middle-aged control group, but lower than that of the young group. The corpus luteum numbers were determined as 14, 5, 9 and 7 for the young, control, NMN and NR groups, respectively (Fig. 4B). The differences in the number of corpus luteum for all groups were statistically important (p < 0.05).

### Analysis of Expression Levels of Mitochondrial Fusion Genes

**Fig. 5:**
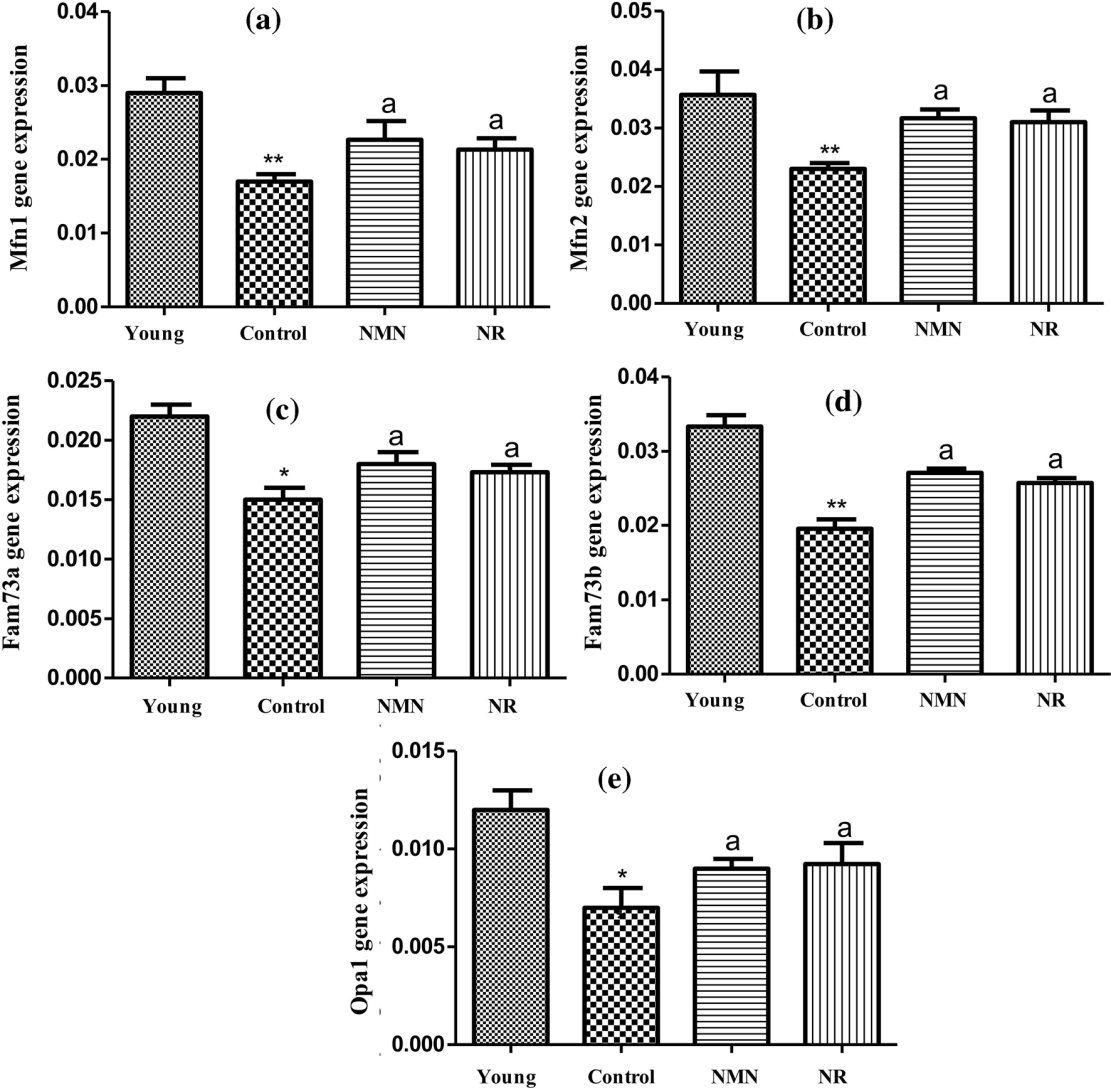
Expression changes of mitochondrial fusion-related genes relative to the β-actin control gene. Mfn1 (**A**), Mfn2 (**B**), Fam73a (**C**), Fam73b (**D**), and Opa1 (**E**). Data were calculated as mean ± standard deviation. *** *p* < 0.001 *vs* Young, ** *p* < 0.01 *vs* young, * *p* < 0.05 *vs* young, a *p* < 0.05 *vs* Control (a *p* < 0.05, b *p* < 0.01, c *p* < 0.001 and n = 3)

The RT-PCR results elucidated that there was a noticable decrease in Mfn1 level of the middle-aged control group in comparison with that of the young group (p < 0.01). The results also revealed that NMN alone or NR alone administration increased significantly Mfn1 levels in comparison with the the middle-aged control group (without NMN and NR). Namely, as compared to the middle-aged control group, noticable increments in Mfn1 levels of the middle-aged NMN and NR groups were determined (p < 0.05). In addition, Mfn1 levels of the NMN and NR groups were found to approach that of Mfn1 of young group (Fig. 5A). As in Mfn1 gene, Mfn2 level of the middle-aged control group also significant decreased in comparison with that of young group(p < 0.01). A statistically higher Mfn2 level was detected in the NMN and NR groups by comparison to the control (p < 0.05). In fact, the Mfn2 levels of the NMN and NR groups were found to be equal almost to the Mfn2 levels of the young group (Fig. 5B). No noticable difference was determined between NMN and NR groups in terms of both Mfn1 and Mfn2 levels (p ≥ 0.05).

The results indicated that there were noticable decreases in Fam73a (Fig. 5C) and Fam73b (Fig. 5D) levels of the middle-aged control group in comparison with the young group (p < 0.05, p < 0.01, respectively). When the effects of NMN and NR applications on Fam73a and Fam73b levels were analyzed, it was seen that both compounds led to noticable increases in Fam 73a and Fam 73b levels compared to the middle-aged control group (p < 0.05). Whereas, no statistical difference was detected between the NMN and NR groups in terms of the expression levels of both Fam73a and Fam73b. (p ≥ 0.05).

The Opa1 level of the control group was ascertained to be statistically lower than that of the young group (p < 0.05). Opa1 levels of NMN and NR groups were found to be higher in a statistical manner than than of the control group (p < 0.05). Besides, it was seen that Opa1 levels of NMN and NR groups approached to that of young group (Fig. 5E). On the contrary, the difference between the Opa1 levels of the NMN and NR groups was not noticable (p ≥ 0.05).

### Analysis of Expression Levels of Mitochondrial Fission Genes

**Fig. 6:**
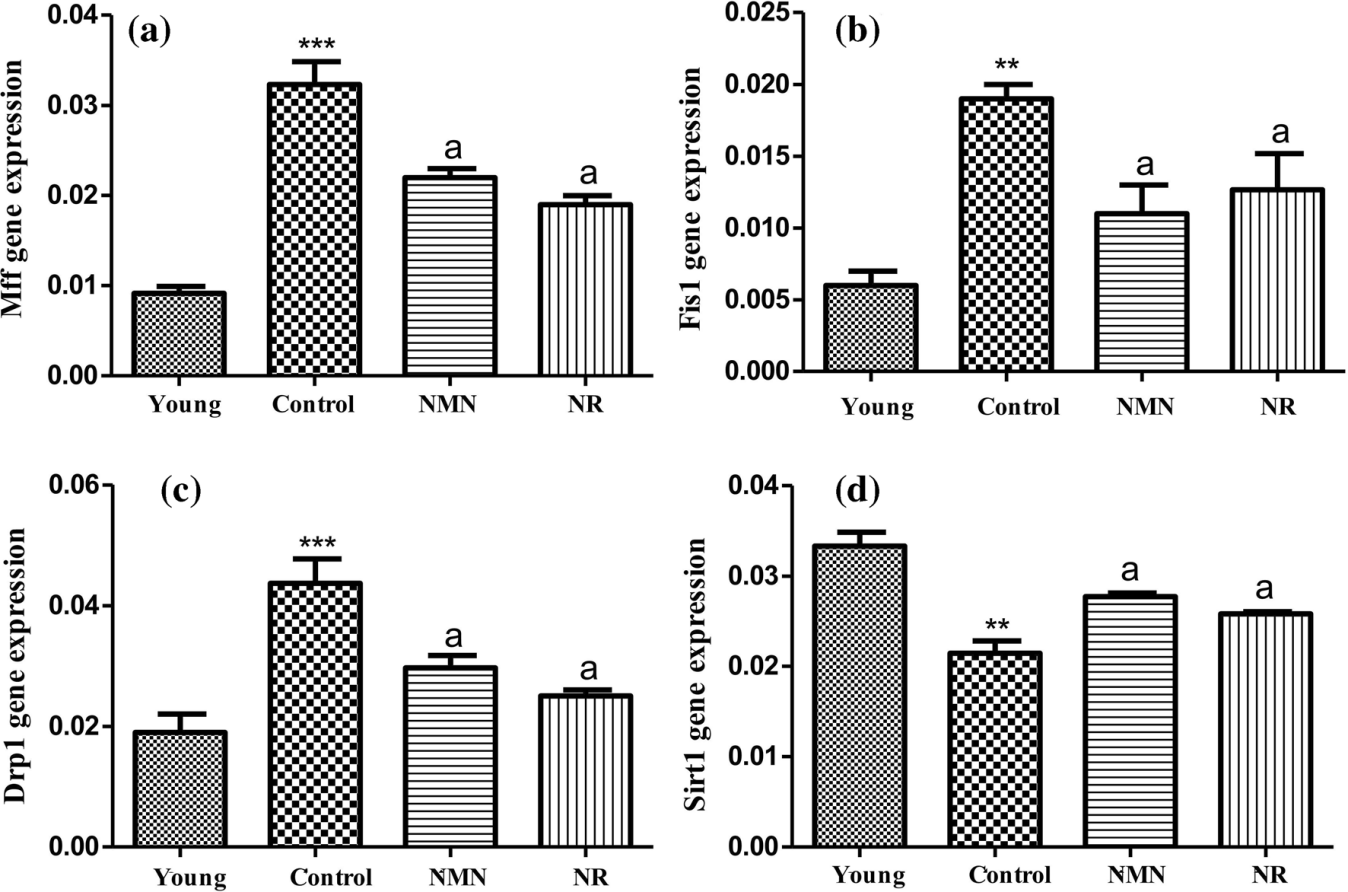
Changes in expression levels of mitochondrial fission-related genes relative to the β-actin control gene. Mff (**A**), Fis1 (**B**), Drp1 (**C**) and Sirt1 (**D**). Data were calculated as mean ± standard deviation. *** *p* < 0.001 *vs* Young, ** *p* < 0.01 *vs* young, * *p* < 0.05 *vs* young, a *p* < 0.05 *vs* Control (a *p* < 0.05, b *p* < 0.01, c *p* < 0.001 and n = 3)

The gene expression analysis demonstrated that there were statistically significant increases in both Mff (Fig. 6A) and Fis1 (Fig. 6B) level of the middle-aged control group when compared with the young group. Especially, the increases in Mff level were higher as compared to Fis1. On the contrary, Fis1 and Mff levels displayed significant decreases in NMN or NR treated groups when compared to the control (p < 0.05). There was no statistical difference between NMN and NR groups in terms of both Fis1 and Mff levels (p ≥ 0.05) (Fig. 6A,B).

The results exhibited that Drp1 expression level of the middle-aged control group was statistically higher than those of other groups (the young group, NMN group, and NR group). Drp1 levels of NMN group and NR group were lower than that of the control group but higher than that of young group. Namely, it was concluded that the application of NMN alone or NR alone downregulated Drp1 level in comparison with the control group but could not completely normalize its level. There was no noticable difference between Drp1 expression levels of NMN and NR groups (p < 0.05).

As seen from Fig. 6D, Sirt1 level of middle-aged control group was lower in a significant manner than that of young group (p < 0.01). A significant increase in Sirt1 expression level was detected in both NMN and NR group compared to the control (p < 0.05). Although NMN alone or NR alone increased Sirt1 level, there was still differences in a significant manner between Sirt1 expression levels of young and NMN group or young and NR group (p < 0.05).

### Analysis of Expression Levels of Fission-Related Proteins by Western-Blot

**Fig. 7:**
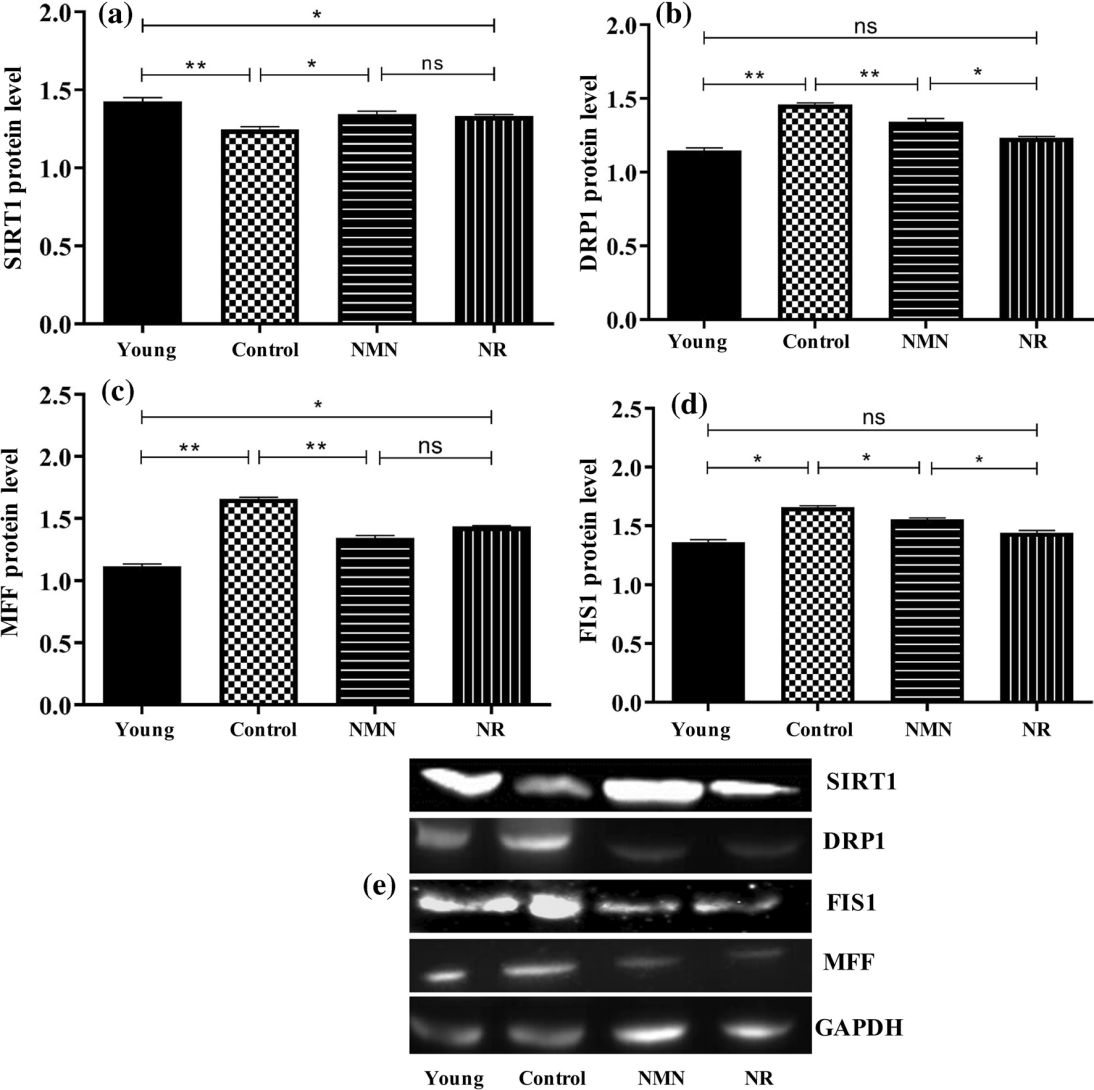
Changes in expression levels of mitochondrial fission-related proteins relative to the GAPDH. SIRT1 (**A**), DRP1 (**B**), MFF (**C**), FIS1 (**D**) and protein bands (**E**). Unpaired t test was applied to the groups. *p* > 0.05 = ns (not significant), *p* < 0.05 = * significant, *p* < 0.01 = * very significant, *p* < 0.001 = ** highly significant

The expression levels of three proteins (MFF, FIS1 and DRP1) directly associated with fission, as well as a protein (SIRT1) indirectly associated with fission, were analyzed by Western Blot (Fig. 7). Compared to the young group, the control group was found to have lower SIRT1 expression levels (p < 0.001). It was found that the SIRT1 levels of NMN and NR groups were higher in a significant manner than that of control group (p < 0.05). SIRT1 levels of NMN and NR groups were found to be lower than that of the young group (p < 0.05). There was no significant difference between SIRT1 levels of NMN and NR groups (p < 0.05) (Fig. 7A and E).

From the results, it became clear that there was a noticable increase in DRP1 level of the middle-aged control group in comparison with the young group (p < 0.05). When compared to the middle-aged control group, a noticable decrease was detected in DRP1 level of both NMN and NR group (p < 0.001, p < 0.05). No significant difference was determined between DRP1 levels of young group and NMN or NR group (p ≥ 0.05) (Fig. 7B and E). As in DRP1, there were statistically significant increases in MFF (p < 0.001) (Fig. 7C and E) and FIS1 (p < 0.01) (Fig. 7D and E) levels of the middle-aged control group in comparison with young group. Especially the expression level of MFF in middle-aged control group was significantly higher than that in the young group. When compared to the control group, there were noticable decreases in MFF and FIS1 levels of NMN or NR group.

## Discussion

Physiological aging is one of the most known reasons of women infertility. While the fertilization success in women is highest level at the age of 20–30, it decreases between the ages of 30–40, and after the age of 40, infertility is observed in one out of every three women [45–48]. During the aging process, both the number and quality of oocytes in the ovaries decreases [18]. For example, the number of primordial follicles (reserve follicles), which is around 400,000 during puberty, decreases to 1000 during menopause due to ovarian aging. Due to the decrease in the number of primordial follicles, menstrual irregularity begins in females and menopause develops in the course of time. The shortening of the menstrual cycle is considered the first sign of decreased ovarian reserve [49–51]. Among the factors contributing to aging, the deterioration of mitochondrial function due to ROS-induced oxidative stress is accepted as one of the most critical factors [52–54].

In this study, the potential beneficial effects of nicotinamide mononucleotide (NMN) and nicotinamide riboside (NR) molecules, which are used as NAD + sources, on folliculogenesis and mitochondrial dynamics in middle-aged rats were investigated.

The results showed that NMN alone or NR alone decreased slightly body weight of middle-aged group. This finding is compatible with the fact that NAD + precursors NMN and NR cause weight loss and prevent obesity [55, 56]. In contrast to middle-aged NMN and NR groups, the average body weight of the young group showed an incremental trend. This was explained by the fact that the rats of the younger group were in the growth stage. The current results also elucidated that the average ovarian weight of the middle-aged control group was higher as compared with the young group. This finding is compatible with the fact that ovarian weight may increase until middle-age period [57]. The ovarian index increased slightly in the NMN and NR groups by comparison with the control. This result indicates that NMN alone or NR alone might have increased ovarian quality, as high ovarian index is accepted as a sign of better female fertility [58].

In the literature, it has been defined that the number of corpus luteum declines with aging and even the corpus luteum disappears completely in rats aged over 12 months [59, 60]. The results of the current study revealed that the administration of NMN or NR increased the number of corpus luteum in middle-aged rats. Today, approaches such as ultrasonographic methods, antral follicle count, ovarian volume and ovarian blood flow measurement are applied in the evaluation of ovarian aging. However, the most widely used test clinically is the antral follicle count because it is easy, inexpensive and reliable [61]. Therefore, the antral follicles were counted to evaluate the degree of ovarian aging in the experimental groups. The data demonstrated that in the middle-aged rats, NMN alone or NR alone increased the number of antral follicles but decreased the number of atretic follicles. Namely, NMN alone or NR alone was determined to improve follicle quality by reducing atresia.

A LH/FSH ratio between 1–2 indicates a healthy ovulation process [62]. Ovarian aging can increase greatly FSH level, but cause a minor decrease in LH level or does not affect [63–65]. Considering the LH/FSH ratios obtained in the present study, it could be deduced that ovarian aging disrupted the LH/FSH balance and increased the atresia of the follicles but NMN or NR application rebalanced LH/FSH ratio and decreased follicular atresia.

Studies have shown that mitochondrial fission and fusion proteins become effective in different processes such as oogenesis, embryogenesis and implantation in mammals [9, 10]. Mfn1, Mfn2 and Opa1 genes have been known to be involved in the fusion of mammalian mitochondria [7]**.** Two other important proteins associated with mitochondria fusion in mammalian are Fam73a and Fam73b. These proteins are known to be responsible for the maintenance of mitochondrial dynamics in ovaries, and in the production and maturation of oocytes in ovaries [9, 10]. The RT-PCR analysis revealed that there were significant reductions in the expression levels of fusion genes (Mfn1, Mfn2, Opa1, Fam73a and Fam73b) of middle-aged rats (control group) in comparison to young group, but NMN or NR increased the expression levels of these genes when applied to middle-aged rats. Even, it was seen that the expression levels of these genes in NMN and NR groups approached the expression levels in young group. The corrective effect of NMN and NR applications on mitochondrial fusion can be considered as an important result. Because, in a study [66], it was stated that Mfn2, a mitochondrial fusion gene, plays a role in development of oocytes and follicles and was necessary for the protection of ovarian follicular reserve.

In mammals, important proteins directly related to mitochondrial fission are DRP1, MFF and FIS1. Of these proteins, DRP1 is located in the cytoplasm, but MFF and FIS1 are positioned in the outer mitochondrial membrane [7, 15, 67]. For the initation of fission, MFF and FIS1 act as receptors and aid the recruitment of DRP1 to the mitochondria. Then, DRP1 forms the ring-like structures around the mitochondria and these rings compress the mitochondria, allowing the mitochondria to divide and the daughter mitochondria to form [68, 69]. The RT-PCR results revealed that Drp1, Fis1 and Mff levels of the middle-aged control group were much higher than the young group. The results are good agreement with the fact that physiological aging disrupts mitochondrial dynamics by enhancing mitochondrial fission in various organs, including the ovaries [25, 26, 70]. The results also showed that when NMN alone or NR alone was applied to middle-aged rats, they caused significant reductions in the expression levels of same fission genes in ovaries. Furthermore, it became clear from the results that there were greater variations in Mff levels as compared with Fis1. This result is consistent with the fact that during fission process Drp1 tends to bind more to Mff than to Fis1, as noted in previous literatures [14, 15, 71, 72]. In case of RT-PCR results, Western Blot results also confirmed that there were noticable increments in the expression levels of the fission proteins (DRP1, MFF and FIS1) in the ovaries of middle-aged-rats, but NMN and NR application significantly diminished the increased levels of these proteins.

Sirtuins have a protective role in delaying ovarian aging in mammals. Especially SIRT1 is involved in the folliculogenesis process in mammals [73–77]. NAD^+^ have a pivotal role in the regulation of the functions of sirtuins. Since sirtuins act as anti-aging enzymes, NAD^+^ deficiency may worsen aging-related diseases by reducing the activity of sirtuins. Therefore, activation of sirtuins by supplementation of NAD^+^ precursors is considered an anti-aging treatment approach [17, 27, 78–80]. For example, in a study [27], NMN administered as a NAD^+^ precursor was shown to improve fertilization and oocyte quality in aged rats. SIRT1 is also related with mitochondrial fission. The action mechanism of SIRT1 is to balance mitochondrial dynamics by reducing the excessive activity of the fission protein DRP1, and thus to alleviate mitochondrial stress [16, 17]. In the current study, RT-PCR analysis displayed that Sirt1 expression was downregulated in the middle-aged group in comparison with the young group. This finding can be explained by the decrease of NAD^+^ level in the ovaries due to aging. However, the results also revealed that Sirt1 level was increased in ovaries of middle-aged rats by NAD^+^ precursor molecules (NMN or NR). In coincide with the RT-PCR results, Western-Blot results also showed that the ovaries of middle-aged rats had a low level of SIRT1 protein, but NMN or NR administration increased the decreased levels of SIRT1 in these rats. Considering all these data, the following hypothesis was developed regarding SIRT1 activity: NAD^+^ released from NAM or NR activated SIRT1, and the activated SIRT1 decreased the high activity of DRP1 and the frequency of mitochondrial fission.

It has been reported in the literature that the use of NMN and NR as drugs or supplements in animal models or humans is safe and does not cause serious side effects even at high doses [33, 81]. For example, Yoshino *et al*. [33] have reported discussed in detail that NMN and NR do not cause toxic effects and exhibit a protective role against many diseases and aging, when applied as oral, intravenous or intraperitonal at doses of 100, 200 and 500 mg/kg in animal models. Cros *et al*. [82] reported that over a 90-day sub-chronic period of repeated oral administration at doses of 375, 750 and 1500 mg/kg/day, NMN appeared to be safe and did not cause toxic effects in Sprague–Dawley rats. Conze *et al*. [83] reported that even if applied not only at a low dose of 300 mg/kg/day but also at high doses of 1000, or 3000 mg/kg/day, NR did not cause a significant toxicity in rats. The present study elucidated that NMN and NR could alleviate ovarian aging in the rats when applied at doses of 500 and 200 mg/kg, respectively. However, higher and lower concentrations of NMN and NR should be tested in future studies in order to fully reveal their roles in alleviating ovarian aging. Moreover, previous studies [33, 81, 84] indicate that the concentrations of NMN or NR tested in animal models are different from the concentrations tested in humans.

## Conclusions

This study displays that ovarian aging in the rats disrupts LH/FSH balance and mitochondrial dynamics (via excess mitochondrial fission) and decreases SIRT1 level as well as the numbers of corpus luteum and antral follicle, but the administration of a NAD^+^ precursor (NMN or NR) restores LH/FSH balance and mitochondrial dynamics, increases SIRT1 activity and alleviates folliculogenesis problems in middle-aged rats. Therefore, we consider that NMN and NR may be used as drug or supplement for reduction of aging-induced folliculogenesis or ovulation problems.

## Data Availability

All data generated or analysed during this study are included in this manuscript.
