# Correction: Nicotinamide Mononucleotide and Nicotinamide Riboside Reverse Ovarian Aging in Rats Via Rebalancing Mitochondrial Fission and Fusion Mechanisms

**DOI:** 10.1007/s11095-024-03716-z

**Published:** 2024-05-16

**Authors:** Nazli Pinar Arslan, Mesut Taskin, Osman Nuri Keles

**Affiliations:** 1 Department of Histology and Embryology, Faculty of Medicine, Ataturk University, Erzurum, Turkey; 2 Vocational School of Health Services, Bingol University, 12000, Bingol, Turkey; 3 Department of Molecular Biology and Genetics, Faculty of Science, Ataturk University, Erzurum, Turkey

**Correction: Pharmaceutical Research**

10.1007/s11095-024-03704-3

The original article has been updated to remove the second affiliation Bingöl University for Osman Nuri Keles. There would be only the 1st affiliation information (Ataturk University) for Osman Nuri Keles. There are no errors in the address information of other authors.

